# Supplementary figures and images for: Predictive value of plasma ephrinB2 levels for amputation risk following endovascular revascularization in peripheral artery disease
Source: PeerJ. 2024 Jun 5;12:e17531. doi: 10.7717/peerj.17531 (PMC11162178; doi:10.7717/peerj.17531)

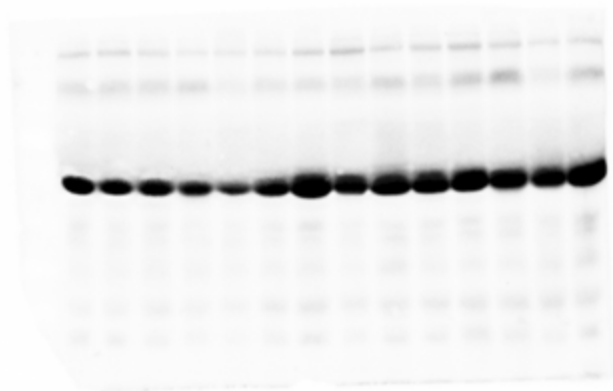

ephrinB2 55kd

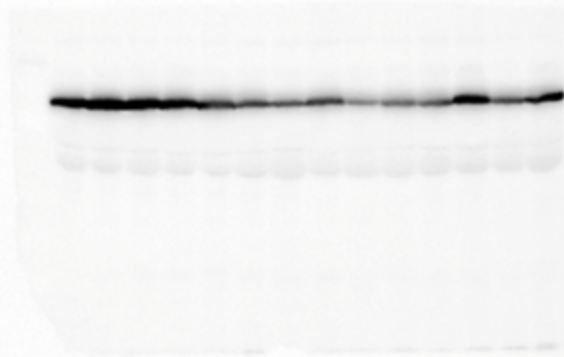

Transferrin 78kd

Supplement: Supplemental Information 1 [file peerj-12-17531-s001.zip › Supplementary 1/Figure 1A.pdf]
