# Supplementary material for: Predictive value of plasma ephrinB2 levels for amputation risk following endovascular revascularization in peripheral artery disease
Source: PeerJ. 2024 Jun 5;12:e17531. doi: 10.7717/peerj.17531 (PMC11162178; doi:10.7717/peerj.17531)
Supplement: Supplemental Information 4 [file peerj-12-17531-s004.docx]

**Codebook**

|  | **0** | **1** |
| --- | --- | --- |
| **Sex** | **Female** | **Male** |
| **Amputation** | **No** | **Yes** |
| **Death** | **No** | **Yes** |
| **hypertension** | **No** | **Yes** |
| **Diabetes** | **No** | **Yes** |
| **CHD** | **No** | **Yes** |
| **COPD** | **No** | **Yes** |
| **Renal Insufficiency** | **No** | **Yes** |
